# Diaphragmatic Hernia Presenting as Acute Gastric Outlet Obstruction: A Rare Complication of Left Lower Lobectomy

**DOI:** 10.7759/cureus.26544

**Published:** 2022-07-04

**Authors:** Anum Sultan, Asma Usman, Sana Akhtar, Anis Rehman, Kashif Siddiqui

**Affiliations:** 1 Radiology, Shaukat Khanum Memorial Cancer Hospital and Research Centre, Lahore, PAK

**Keywords:** thoracotomy, computed tomography, lobectomy, acute gastric oulet obstruction, iatrogenic diaphragmatic hernia

## Abstract

Diaphragmatic hernia is defined as the prolapse of abdominal contents into the thoracic cavity through a defect in the diaphragm that is either congenital or acquired. Acquired hernias are common in adults and frequently occur as the result of trauma, either iatrogenic or non-iatrogenic. Iatrogenic diaphragmatic hernia is a rare complication of patient-related treatment maneuvers/procedures. The rate of late presentations of an iatrogenic diaphragmatic hernia is disparate, ranging from 5 to 62%. Iatrogenic diaphragmatic hernia after pulmonary resection is extremely rare with only two case reports published worldwide so far. In this report, we discuss the case of a young male presenting several years after undergoing left lower lobectomy with signs and symptoms of acute gastric outlet obstruction.

## Introduction

Diaphragmatic hernia is a condition characterized by the prolapse of abdominal contents through a defect in the diaphragm into the thoracic cavity. Diaphragmatic hernia can be congenital or acquired [1]. Most commonly, diaphragmatic hernias are congenital, accounting for more than 80-90% of all cases [1]. These develop as a result of the failure of the closure of the pleuro-peritoneal membrane during embryonic development. Acquired hernias are common in adults and frequently occur as a result of trauma [2], either iatrogenic or non-iatrogenic [3]. Most traumatic diaphragmatic injuries result from road traffic accidents (80-85%) or penetrating injury to the lower chest wall (10-15%) [4,5].

Iatrogenic diaphragmatic hernia is a rare complication of patient-related treatment maneuvers/procedures such as surgery, chest tube misplacement, and, in rare cases, cardiopulmonary resuscitation causing accidental trauma to the diaphragm [3]. Most surgical injuries are usually diagnosed and repaired intraoperatively; however, some of them can be missed and present months and years after the initial trauma [3]. The rate of a delayed presentation of an iatrogenic diaphragmatic hernia ranges from 5 to 62% [3]. The longest delay in presentation has been reported to be 35 years [6]. In the literature, Iatrogenic diaphragmatic hernias have been reported following esophagectomy [7], gastrectomy [8], laparoscopic cholecystectomy [9], hepatectomy [3], and nephrectomy [10,11]. Iatrogenic diaphragmatic hernia after pulmonary resection is extremely rare with only two case reports published worldwide: by Pan et al. in 2016 and Hong et al. in 2017 [11,12].

The clinical presentation of diaphragmatic hernia is variable, ranging from asymptomatic to respiratory, cardiac, and bowel-related complications. Depending on the involved organ, mortality rates can reach as high as 25% [13,14]. An acute presentation with a strangulated or an obstructed viscus may pose a diagnostic dilemma and require urgent resection and repair [11]. We report the case of a young male patient presenting several years after left lower lobectomy with signs and symptoms of acute gastric outlet obstruction.

## Case presentation

The patient was a 47-year-old male who presented to the emergency department with complaints of multiple episodes of vomiting for four days associated with abdominal pain and passage of a small amount of stool and flatus every day. He had a history of right testicular yolk sac tumor for which he had undergone orchidectomy five years ago. Later on, three years ago, he had presented with pulmonary metastasis and had undergone left thoracotomy with the findings of a large left lower lobe mass adherent to the diaphragm, which had been resected along with a small portion of the left diaphragm. Wedge resection of the left upper lobe metastatic deposit had also been performed. The diaphragmatic defect had been repaired with a Prolene suture. The patient had experienced an uneventful recovery and had then been discharged.

On examination, his abdomen was soft, non-tender, with no mass palpable, and not distended; succession splash was negative and his digital rectal examination showed some stool in the rectum with normal consistency. His blood pressure was 130/90 mmHg, respiratory rate was 14 breaths/minute, pulse rate was 80/minute, oxygen saturation was 98%, and he was afebrile. Laboratory investigations are presented in Table 1.

**Table 1:** Baseline investigations WBC: white blood cell; RBC: red blood cell; MCV: mean corpuscular volume; MCH: mean corpuscular hemoglobin; MCHC: mean corpuscular hemoglobin concentration; RWD-CV: red cell distribution width - coefficient of variation; CRP: C-reactive protein; INR: international normalized ratio; GFR: glomerular filtration rate

Investigations	Results	Units	Normal range
Complete blood picture			
WBC	10.7	10^3^/ul	4-10
RBC	5.81	10^6^/uL	4.5-5.5
Hemoglobin	13.2	g/dL	13-17
Haematocrit	42.1	%	40-50
MCV	31.4	g/dL	76-96
MCHC	31.4	g/dL	31.5-34.5
MCH	22.7	pg	27-32
RDW-CV	15.1	%	11.5-14.5
Platelets	294	10^3^/uL	150-450
Neutrophils	65.6	%	40-80
Lymphocytes	26.6	%	20-40
Monocytes	6.9	%	2-10
Eosinophils	0.7	%	1-6
Basophils	0.2	%	<1
CRP	5.4	mg/L	<5
Lactate	12.4	mg/dL	4.5-19.8
Coagulation profile			
Prothrombin time	11.1	Seconds	9-14
INR (calculated value)	1.05		
Serum electrolytes			
Sodium	142	mmol/L	136-145
Potassium	4.09	mmol/L	3.5-5.1
Chloride	104.1	mmol/L	98-107
Bicarbonate	21.9	mmol/L	22-29
Urea nitrogen	7.9	mg/dL	6-20
Creatinine	0.71	mg/dL	0.70-1.20
eGFR	118.85	mL/min/1.73 m^2^	<60

**Figure 1:**
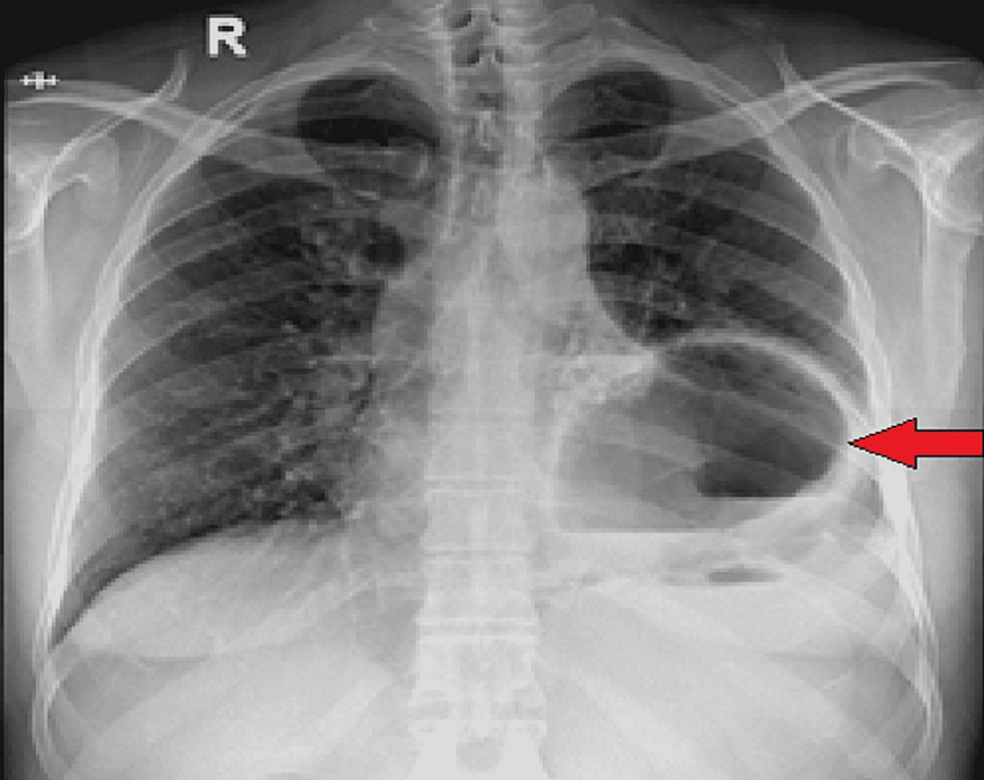
X-ray chest AP view showing a cystic area in left hemithorax demonstrating air-fluid level (red arrow) AP: anteroposterior

A chest X-Ray was performed, which showed cavitation with the air-fluid level in the left lower thorax (Figure 1). The abdominal X-ray showed a nonspecific bowel gas pattern with no free air under the diaphragm. Impression of obstructed hiatal hernia was made and a surgical team was taken on board.

**Figure 2:**
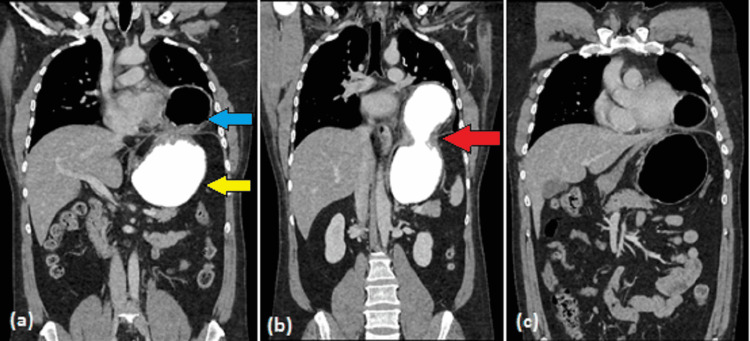
CT scan coronal images with intravenous and oral contrast The images show a left diaphragmatic defect (red arrow) with herniation of the proximal stomach (blue arrow) into the left hemithorax. The distal part of the stomach is seen in its anatomical position in the left upper abdomen (yellow arrow) CT: computed tomography

An abdominal CT was also performed, which showed a left diaphragmatic defect measuring 1.9 cm with herniation of the distal part of the stomach through the defect into the left hemithorax. There was the passage of contrast through the afferent loop into the distal stomach; however, the passage of contrast was not seen through the efferent loop into the duodenum (Figure 2). Delayed images were taken after one hour with unchanged appearances. There was an associated left lower lobe atelectasis. No contrast leakage was noted. There was no pneumoperitoneum. The findings were suggestive of a diaphragmatic hernia with gastric outlet obstruction.

**Figure 3:**
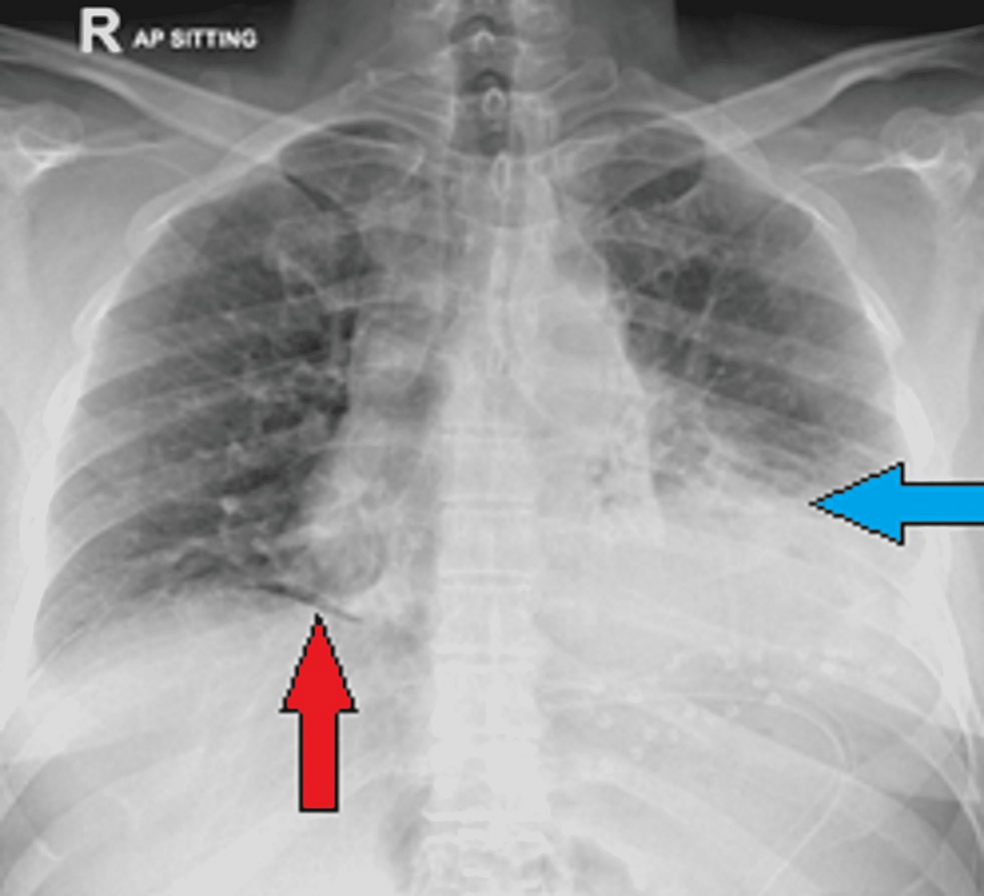
Postoperative X-ray chest showing left lower zone atelectatic changes Traces of post-surgical pneumoperitoneum are seen below the right hemidiaphragm

The patient then underwent laparoscopy, which revealed a 5 x 5-cm defect in the left diaphragm with body and cardia of stomach herniated through it into the left hemithorax. The rest of the stomach was viable with no serosal tear or any other defect; working ports were inserted but, due to dense adhesions, the reduction was difficult and the procedure was converted to open laparotomy. An upper midline incision was performed. Adhesiolysis was undertaken and the stomach was reduced through the defect. The diaphragmatic defect was closed using Prolene mesh; drains were placed and the incision was closed with Prolene sutures. Postoperatively, he was shifted to the ICU. Follow-up chest X-ray showed minimal postoperative pneumoperitoneum and left basilar atelectasis (Figure 3). The patient stayed in the ICU for two days with an uneventful recovery and was later discharged after five days.

## Discussion

Iatrogenic diaphragmatic hernia is an infrequent entity with only a few cases reported in the literature so far [4]. Delayed presentation is rare, resulting in difficulty in diagnosis, thereby leading to significant morbidity and mortality [4]. This necessitates obtaining a detailed clinical history, with particular emphasis on past surgical procedures and interventions, accompanied by a meticulous clinical examination.

It is believed that small injuries to the diaphragm may occur preoperatively due to strong adhesions between the tumor and diaphragm. The failure to promptly recognize these defects leads to the gradual increase in their size over the years due to the pressure gradient between abdominal and pleural cavities, leading to delayed presentation in the form of a diaphragmatic hernia [12].

Various radiological investigations such as chest and abdominal X-rays, upper gastrointestinal tract (UGI) fluoroscopy with oral contrast, and CT scan can help in establishing the diagnosis. A chest X-ray usually provides a preliminary clue to the disease. CT scan is the imaging modality of choice in the characterization and identification of diaphragmatic hernias as well as their complications [15] with a sensitivity of 55% and specificity of 100% [3].

Our patient had metastatic pulmonary disease adherent to the diaphragm, which was resected, and the diaphragmatic defect was repaired during the surgery. This emphasizes the fact that the integrity of the diaphragmatic dome needs to be assessed at the end of the surgery as well as on follow-up imaging in patients with prior thoracotomy and tumor resection. Coronal and sagittal images are particularly useful in this regard as the diaphragm gets outlined more clearly on these reformatted images than on axial images. Furthermore, attention should be paid to the patient’s symptoms of abdominal pain, vomiting, dyspnea, and altered bowel habits during follow-up visits after surgery as early recognition of bowel obstruction can prevent serious complications. Surgical reduction laparoscopically or laparotomy is the treatment of choice and should be expedited in cases of acute obstruction.

## Conclusions

Iatrogenic diaphragmatic hernia is a rare but known complication after thoracotomy, which needs to be considered in postoperative patients presenting with signs and symptoms of bowel obstruction. Early recognition and timely intervention can prevent grave complications such as volvulus, strangulation, incarceration, ischemia, and perforation. Surgery is the mainstay of treatment with a pivotal role in reducing morbidity and mortality.
